# Cardiac Imaging in Liver Transplantation Candidates: Current Knowledge and Future Perspectives

**DOI:** 10.3390/jcm8122132

**Published:** 2019-12-03

**Authors:** Yannis Dimitroglou, Constantina Aggeli, Alexandra Alexopoulou, Sophie Mavrogeni, Dimitris Tousoulis

**Affiliations:** 1 Department of Cardiology, National and Kapodistrian University of Athens Medical School, Hippokration General Hospital, 115 27 Athens, Greece; dina.aggeli@gmail.com (C.A.); drtousoulis@hotmail.com (D.T.); 2 Department of Internal Medicine and Research Laboratory, National and Kapodistrian University of Athens Medical School, Hippokration General Hospital, 115 27 Athens, Greece; 3 Onassis Cardiac Center and National and Kapodistrian University of Athens, 176 74 Athens, Greece; sophie.mavrogeni@gmail.com

**Keywords:** liver cirrhosis, cirrhotic cardiomyopathy, cardiac imaging, echocardiography, cardiac magnetic resonance, liver transplantation

## Abstract

Cardiovascular dysfunction in cirrhotic patients is a recognized clinical entity commonly referred to as cirrhotic cardiomyopathy. Systematic inflammation, autonomic dysfunction, and activation of vasodilatory factors lead to hyperdynamic circulation with high cardiac output and low peripheral vascular resistance. Counter acting mechanisms as well as direct effects on cardiac cells led to systolic or diastolic dysfunction and electromechanical abnormalities, which are usually masked at rest but exposed at stress situations. While cardiovascular complications and mortality are common in patients undergoing liver transplantation, they cannot be adequately predicted by conventional cardiac examination including transthoracic echocardiography. Newer echocardiography indices and other imaging modalities such as cardiac magnetic resonance have shown increased diagnostic accuracy with predictive implications in cardiovascular diseases. The scope of this review was to describe the role of cardiac imaging in the preoperative assessment of liver transplantation candidates with comprehensive analysis of the future perspectives anticipated by the use of newer echocardiography indices and cardiac magnetic resonance applications.

## 1. Introduction

The effects of end stage liver disease in the function of the cardiovascular system are a recognized clinical entity frequently referred to as cirrhotic cardiomyopathy (CCM) [1]. CCM is characterized by a high output state at rest with impaired ability to increase contractility at stress, diastolic dysfunction, and electromechanical abnormalities [2]. Cardiac dysfunction is frequently masked because low peripheral resistances induced by arterial vasodilation of the splanchnic circulation decrease left ventricular afterload and thus cardiac work. However, when the heart is exposed to an acute event such as infection, gastrointestinal bleeding or after transjugular intrahepatic portosystemic shunt (TIPS) procedure, the reduced response to adrenergic stimulation may unmask cardiac dysfunction resulting in heart failure [3]. Liver transplantation (LT) itself may also unmask cardiac dysfunction leading to severe manifestations of heart failure [4] and as many as 50% show signs of cardiovascular dysfunction in the first week after LT [5]. More specifically, severe acute pulmonary edema has been reported in 9% of liver transplant recipients following surgery, hemodynamically significant arrhythmias in 6% and new onset atrial fibrillation in 10% [6,7,8]. Consequently, up to 21% of deaths following LT can be attributed to heart failure [9] and up to 40% to cardiovascular disease in general [10]. Other complications of end-stage liver disease include hepatorenal syndrome, hepatopulmonary syndrome, and portopulmonary hypertension which may also affect cardiac function [11,12,13]. For those reasons and according to the European Association for the Study of the Liver guidelines, transthoracic echocardiography is required in all LT candidates and in selected cases, stress echocardiography should be performed for preprocedural evaluation and risk stratification [14].

However, CCM often remains unrecognised when using traditional cardiovascular imaging modalities [15] and heart failure can be observed after LT even after all preoperative tests are negative [16]. Thus, newer more sensitive echocardiography indices as well as other imaging modalities, such as Cardiac Magnetic Resonance (CMR), are needed to improve the diagnostic approach of CCM, portopulmonary hypertension and other complications of end-stage-liver-disease.

## 2. Pathophysiological Aspects of the Cardiovascular Function of Cirrhotic Patients

**Figure 1:**
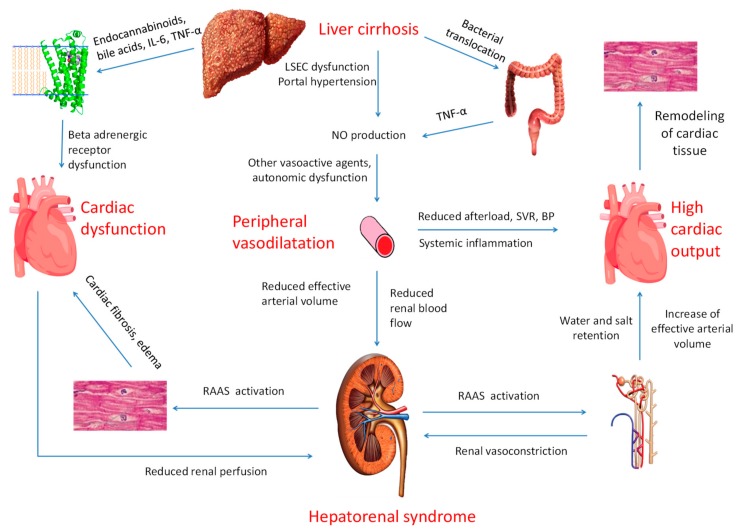
Presentation of pathophysiological interactions between the liver the heart and the kidneys for the pathogenesis of cirrhotic cardiomyopathy. RAAS: renin-angiotensin-aldosterone system, LSEC: liver sinusoidal endothelial cells.

Cardiac manifestations in patients with advanced cirrhosis are a result of heart remodeling to compensate for the high output state and of the direct effects in the cardiac cell (Figure 1).

Changes in systematic circulation have been related with changes in hepatic blood flow and portal pressure [17]. Dysfunction of liver sinusoidal endothelial cells (LSEC) contribute to an increase in hepatic vascular resistance and portal hypertension [18]. They also trigger signals which induce increased production of factors such as vascular endothelial growth factor and angiotensin-(1―7) [19,20]. These factors facilitate the formation of collateral portosystemic vessels and also lead to an increase of nitric oxide synthase (eNOS) and a subsequent peripheral vasodilation [21]. Nitric oxide production is also facilitated by bacterial translocation and inflammatory molecules, such as TNF-α [22]. Other vasodilatory molecules include endocannabidoids, carbon monoxide, adrenomeduline, and prostacycline [22,23]. The sympathetic nervous system is activated, but vascular smooth muscle cells cannot respond properly to adrenergic stimuli or vasoconstrictive molecules [23,24]. Moreover, plasma volume in the splachnic circulation or extravascular space is expanded. As a result, decreased effective arterial blood volume and blood pressure can lead to a reduction in renal perfusion and an increase in renin production [25]. These alterations and angiotensin-(1―7) production result to renin-angiotensin-aldosteron system (RAAS) activation and changes in volume status (high output state) and electrolyte balance [26]. High output state irrespective of etiology, has been shown by Reddy et al. to induce left ventricular remodeling including increased ventricular dimensions, increased left ventricular mass, right atrial pressure and diastolic dysfunction [27]. When compared to other diseases, cirrhotic patients seem to have lower peripheral resistance and blood pressure factors associated with worse prognosis.

Direct effects on the cardiac cells of cirrhotic patients are mediated mainly through the down-regulation and desensitization of b-adrenergic receptors [28]. Endocannabinoids, bile acids, and inflammatory agents have both direct negative inotropic effects or indirect through the desensitization of beta-adrenergic receptors [25,29]. It is also believed that RAAS mediated fibrosis and edema contributes to impaired left ventricular relaxation and diastolic dysfunction [30]. Desensitization of beta-adrenergic receptors, inflammation-induced cardiodepression, and diastolic dysfunction can lead to a blunted response during stress and to the development of emergent heart failure symptomatology [25,29].

According to the Montreal Criteria introduced in 2005 (Table 1), CCM is characterized by impairments of systolic or diastolic function and additional elements such as absence of electromechanical coordination [5]. However, based on newer knowledge about CCM pathophysiology and clinical significance, voices have been raised about a possible reconsideration of diagnostic criteria [15]. To this respect, the incorporation of newer cardiac imaging modalities in these criteria should be applied based on emerging clinical data.

According to an inspection of 639 autopsies of cirrhotic patients compared to over 17,000 autopsies of the general population, myocardial infarction was about four times lower in cirrhotic patients (4.9%) when compared to other populations (20.2%) [31]. These findings were later explained by the low cholesterol values and low blood pressure [32]. However, newer data have shown that coronary artery disease (CAD) in cirrhotic patients is not as rare as it was considered to be. Incidence of CAD in patients evaluated for LT has been found 26% in coronary angiography [33]. According to Keeling et al., who performed a multidetector computed tomography coronary angiography, 34% of cirrhotic patients during LT evaluation had moderate or severe CAD [34]. Several factors are believed to have contributed to these changes. Patients with non-alcoholic steatohepatitis (NASH) associated cirrhosis often have dyslipidemia or diabetes mellitus which are traditional risk factors for CAD. Frequency of CAD in patients with NASH-cirrhosis has been found to be significantly higher than in other cirrhotic patients [31]. Because of the increased frequency of metabolic syndrome and non-alcoholic fatty liver disease [35], frequency of NASH cirrhosis has increased leading to increased CAD prevalence in cirrhotic patients [36].

## 3. Cirrhotic Cardiomyopathy Diagnostic Approach with Echocardiography: Current Knowledge

Quantification of cardiac chamber dimensions using standard two-dimensional (2D) echocardiography has revealed an increase of left ventricular end diastolic volume and left atrial volume index in cirrhotic patients which is correlated with the disease severity and the Model for End stage Liver Disease (MELD) score [37]. Right heart may also be affected as shown in an early study by Pozzi et al. [38]. Moreover, left ventricular hypertrophy may also be apparent and has been correlated with worse prognosis after LT probably because of impaired myocardial relaxation [38].

Because of the high output state and reduced afterload, systolic function, as can be assessed using left ventricular ejection fraction (LVEF), is found normal or even increased in most patients [39]. LVEF is not a sensitive marker of myocardial dysfunction in cirrhosis. However, the reduced value should be taken into account because it has been associated with bad prognosis in cirrhotic patients before and after LT [9]. Systolic dysfunction in patients with alcoholic etiology of cirrhosis can be attributed to alcoholic cardiomyopathy, but in such scenarios, systolic function typically improves after alcohol withdrawal [40]. Other than that, significant cardiac dysfunction due to alcoholic cardiomyopathy is rarely seen in LT candidates, because most patients have abstained from alcohol for at least 6-months before LT [14].

Traditional indices of diastolic dysfunction such as early diastolic mitral inflow velocity/ late diastolic mitral inflow velocity (E/A ratio), isovolumetric relaxation time and deceleration time have been used as diagnostic criteria of cirrhotic cardiomyopathy [15]. E/A ratio is not a specific marker and its assessment may be affected by loading conditions. So a decrease in E/A ratio may be precipitated by the high output state and does not always indicate diastolic dysfunction in cirrhotic patients. For this reason, indices of left ventricular diastolic dysfunction which are relatively preload independent, such as tissue Doppler imaging (TDI)-derived mitral annulus velocity during early wave (e’), as well as E/e’ ratio are superior to the conventional Doppler echocardiographic measurements as predictors to outcome in cirrhotic patients [41,42]. In a recently published systematic review, diastolic dysfunction grade 2 was shown to be correlated with the worst prognosis [43]. The detrimental effect of diastolic dysfunction in the prognosis of cirrhotic patients has also been highlighted by studies displaying decreased survival in patients with increased left ventricular mass index (LVmassi) and left atrial volume index (LAVi) [38,44]. However, it is not clear whether LVmassi and LAVi behave as independent variables of survival or they are disturbed in advanced stages of liver disease as a result of the remodelling induced by the high output state.

Heart failure in LT recipients is believed to be the result of non-ischemic systolic dysfunction [45]. A decrease of EF < 40% can be found in up to 11.7% of patients undergoing LT within six months after the procedure [46]. Even though a clear ischemic etiology is not diagnosed in most of those patients, preprocedural segmental wall-motion abnormalities are predictive of deterioration post-transplantation, probably indicating microcirculation disturbances. Moreover, while diastolic dysfunction is a predictive factor of heart failure after LT [45], prognostic role of newer advanced indices of diastolic dysfunction has yet to be examined.

## 4. Cirrhotic Cardiomyopathy Diagnostic Approach with Echocardiography: Future Perspectives

Myocardial Performance Index (MPI/Tei index) is defined as the ratio of isovolumetric contraction time plus isovolumetric relaxation time to the ejection time and has found to be increased in heart failure patients [47]. It has also been shown to be an early marker of systolic or diastolic dysfunction [48,49] and correlates with disease severity [50] and adverse prognosis [51,52]. TDI-derived Tei index is considered more reliable and reproducible than that calculated using pulse-wave Doppler to assess global cardiac function [50]. This index has been assessed in few studies with cirrhotic patients and has been demonstrated to be related with cirrhosis severity and brain natriuretic peptide (BNP) levels [53,54]. The increase of Tei index may be considered a sensitive marker of the inability of the left ventricle to further increase cardiac work in advanced cirrhosis or as a response to adrenergic stimuli in cirrhotic patients. As Tei index is affected by systolic and diastolic function [55] as well as afterload and ventriculoarterial coupling [56], further studies evaluating its prognostic role are needed.

Speckle tracking echocardiography (STE) is based on the generation of small myocardial footprints and frame by frame identification of their relative displacement which enables quantification of myocardial deformation [57]. Global longitudinal strain (GLS) is considered an early marker of systolic dysfunction affected earlier than the LVEF. Moreover, it is also disturbed in patients with heart failure and preserved LVEF [58] or in patients with hypertrophic cardiomyopathy [59]. A Comparative study of STE and cardiovascular magnetic resonance (CMR) have shown impaired GLS in myocardial segments with extended fibrosis [59]. A few studies have used STE to assess myocardial function in cirrhotic patients with conflicting results. In some research works absolute GLS values are lower than in controls [60,61], while in others cirrhotic patients had GLS values within the normal limits [62] and without significant differences compared to controls [63]. Even though GLS seems not to be affected by cirrhosis severity and diastolic dysfunction grade [64], there are limited data in the literature to pinpoint an increase in absolute GLS after LT [61].

**Figure 2:**
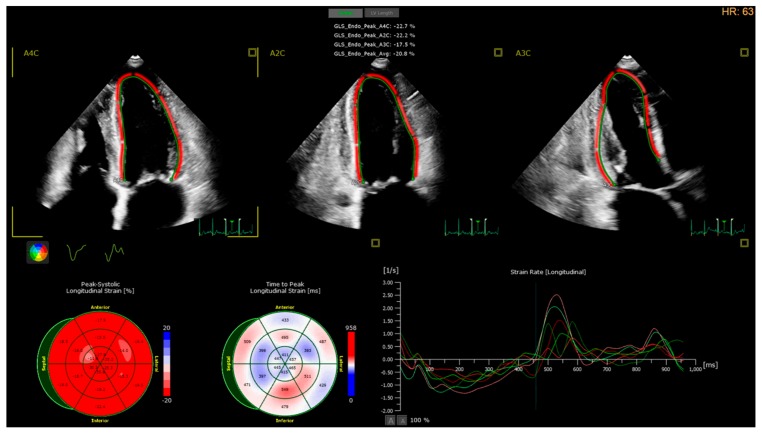
Speckle tracking echocardiography. Semiautomatic calculation of Global longitudinal strain (GLS), with possible simultaneous identification of segmental wall motion abnormalities (left low), dysychrony (middle low) and diastolic function abnormalities-strain rate (right low).

Except from GLS, STE can be useful in the diagnosis of diastolic dysfunction. Strain rate during isovolumetric relaxation and early diastole are related with LV filling pressure and wedge pressure and may be sensitive markers of impaired LV relaxation [65]. To our knowledge only one study has used the strain rate to evaluate cirrhotic patients [63]. Left atrial strain is a marker not only of LV filling pressures [65], but also a marker of atrial function and thus has an additive value when diagnosing and determining severity and prognosis of patients with diastolic dysfunction [66]. We believe that thorough evaluation of cardiac function in cirrhotic patients using STE will eventually lead to a better CCM characterization, better explanation of pathophysiology mechanisms and the identification of predictive and prognostic markers in LT candidates (Figure 2).

**Figure 3:**
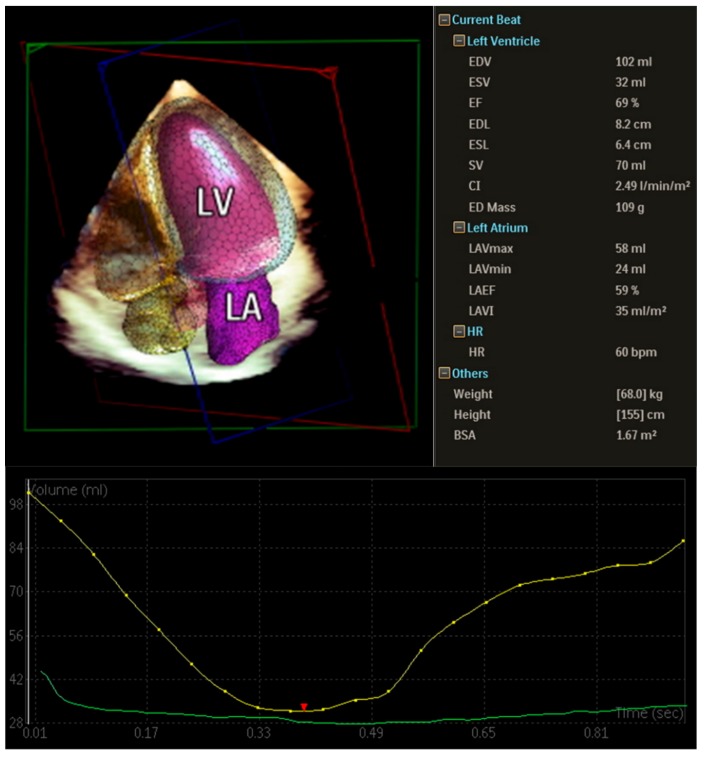
3D echocardiography. Semi-automatic calculation of cardiac chamber dimensions, systolic function, atrial function, and left ventricular mass.

Three dimensional (3D) echocardiography is helpful when quantifying volume and function of cardiac chambers, is considered more accurate and more reproducible than 2D echocardiography and may also permit more accurate measurement of LV mass [67]. Those advantages are the result of eliminating the need of geometrical assumptions which are obligatory in 2D measurements and M-mode calculation of LV mass. However, poor acoustic window and difficulty to maintain a steady body position or stop breathing in patients with advanced cirrhosis and large ascites decrease the feasibility of this imaging modality (Figure 3).

Contrast echocardiography utilizing contrast agents and low mechanical index imaging modalities (left ventricular opacification-LVO) can offer many advantages on the evaluation of cardiac dysfunction in cirrhotic patients. Differentiation of ventricular cavity to myocardium enables accurate detection of endocardial borders and recognition of segmental wall-motion abnormalities [68]. Furthermore, contrast echocardiography can attenuate the limitations of poor acoustic window.

## 5. Stress Echocardiography in LT Candidates. Current Knowledge

A distinctive feature in CCM is the inability to increase cardiac work at stress. Thus, stress echocardiography (SE) is used first to reveal the blunted response to adrenergic stimulation and secondly to diagnose CAD. Several studies have been published using either exercise test or dobutamine induced stress to evaluate the diagnostic and prognostic role of SE in cirrhotic patients and/or LT candidates.

Blunted response to adrenergic stimulation is translated into lower peak heart rate, lower increase in LVEF and cardiac index in cirrhotics compared to controls during peak exercise [69]. These findings result from the autonomic dysfunction characterizing end-stage cirrhosis [70]. Besides that, blunted response during dobutamine stress echo has been accompanied by higher baseline ejection fraction [71], a finding which highlights that contractile reserve is lower in patients with decompensated cirrhosis and high output cardiovascular state. Some data with the use of STE reinforce these findings. In a recently published study, while GLS at rest was better in cirrhotics than controls, groups did not differ at stress, because lower absolute GLS increase was observed in cirrhotic patients [72]. However, due to temporal resolution limitations, use of speckle tracking echocardiography in current clinical practice of stress echocardiography is questionable and more data from future studies are needed.

According to a meta-analysis which evaluated the diagnostic accuracy of dobutamine SE in diagnosing hemodynamically significant coronary artery stenoses, there is a high heterogeneity among studies, demonstrating low sensitivity of 28% and high specificity of 82% [73]. However, in most of those studies only a small proportion of the patients underwent coronary angiography. Low sensitivity has also been found in a larger scale study published recently. In this investigation, 633 patients who had undergone both dobutamine SE and coronary angiography were included [74]. Sensitivity was calculated at 24%, meaning that less than one out of four patients with hemodynamically significant coronary artery stenosis, had a positive SE. Given that CAD was documented invasively in only 12% of the patients, positive predictive value was also low. Those findings can be explained at part because goal heart rate during dobutamine SE is achieved in less than 80% of LT candidates either because of autonomic dysfunction or because of the concomitant use of beta-blockers. In this study, lack of hyperkinisis at high-dose stages of the protocol as well as presence of tardokinesis increased the diagnostic accuracy of the SE. Nevertheless, ischemic response during dobutamine SE by itself does not seem to have a high predictive value for cardiac events and cardiac death after LT [75,76]. For those reasons, simultaneous evaluation of contractile, chronotropic as well as ischemic response may increase diagnostic accuracy of SE protocols.

## 6. Stress Echocardiography in LT Candidates. Future Perspectives

Extended use of beta blockers and autonomic dysfunction attenuates the ability of dobutamine to increase heart rate and accomplish a diagnostic SE. Even though there are limited data showing that the maximum heart rate achieved may inversely correlate with adverse events [77], the prognostic value of non-diagnostic tests in LT candidates remains to be more thoroughly evaluated. Moreover, although coronary angiography is accurate when diagnosing hemodynamically significant stenoses, it cannot determine microcirculation disturbances [78]. Microcirculation disorders are common in CCM models and are precipitated by inflammatory mechanisms [79], while patients with NASH-associated cirrhosis and diabetes possibly have more extended microcirculation abnormalities [80]. Thus, it is possible that a stress echo considered false positive after coronary angiography may actually point towards unrecognized microcirculation disorders.

During vasodilator studies, ischemia is induced by coronary steal of blood flow away from the stenotic artery [81] and thus, oxygen delivery is not determined by heart rate and cardiac work increase [82]. For this reason myocardial perfusion studies using scintigraphy have shown higher sensitivity of 62% in diagnosing hemodynamically significant CAD, but with lower specificity [73]. However, with perfusion studies, autonomic dysfunction and blunted adrenergic response cannot be evaluated even though they are the hallmark of cirrhotic cardiomyopathy. Consequently, perfusion studies during dobutamine SE protocols can yield significant advantages.

When performing dobutamine SE with contrast agents, myocardial perfusion can be evaluated with quantitative or semi-quantitative methods using a combination of high and low mechanical index imaging modalities [83]. This can be achieved in the same examination where wall motion and contractile response can also be estimated. Baibhav et al. who examined the prognostic significance of abnormal wall motion and myocardial perfusion in LT candidates, found that patients with abnormal perfusion during dobutamine SE have a 7-fold increase of the risk for cardiovascular event following LT [84]. In the multivariate analysis, it was also found that the presence of myocardial perfusion abnormalities was the only independent predictor of adverse outcome. As dobutamine SE protocols maintain the disadvantage that goal heart rate is more difficult to be achieved in cirrhotic patients, we believe that mixed protocols including a combination of dobutamine and vasoactive agents such as adenosine or regadenoson will prove to have higher diagnostic accuracy for obstructive CAD and higher prognostic value for cardiovascular outcome after LT.

Diastolic SE with supine bicycle reveals worsening of diastolic dysfunction in patients with reduced diastolic reserve and can possibly exacerbate symptoms of heart failure when they are masked at rest [85]. During diastolic SE, parameters like diastolic transmitral flow (E, A waves), mitral annulus TDI indices (e’), and systolic pulmonary artery pressure are measured and their alterations with values at rest are evaluated [86]. This can be accomplished in parallel with contractile reserve and ischemic response evaluation. Given the frequency, the prognostic significance and the correlation of diastolic dysfunction with cardiac events in LT candidates, we believe that a study evaluating diastolic SE feasibility and prognostic utility should be designed. When physical exercise is feasible, supine bicycle can be used for cardiopulmonary exercise testing (CPET) which is an objective test to assess functional status and frailty factors with high prognostic significance [87]. According to a meta-analysis by Ney et al., CPET findings and especially ventilator anaerobic testing can predict pre and post-transplantation survival in cirrhotic patients [88]. However, given the high heterogeneity of published studies, more scientific data are needed before CPET is incorporated in the clinical practice.

## 7. Hepatopulmonary Syndrome and Portopulmonary Hypertension: Current Knowledge

Hepatopulmonary syndrome (HPS) is caused by intrapulmonary vasodialation or arteriovenous communications and leads to hypoxia because of ventilation/perfusion (V/Q) mismatch [89]. Common symptoms are dyspnea and platypnea with orthodeoxia. Orthodeoxia (decrease in oxygen saturation while upright or in siting body position) is a characteristic sign and can be justified by the increased V/Q mismatch. HPS can be detected in about 20% of cirrhotic patients and up to 50% of end-stage patients undergoing LT evaluation but those values differ across the studies [90].

**Figure 4:**
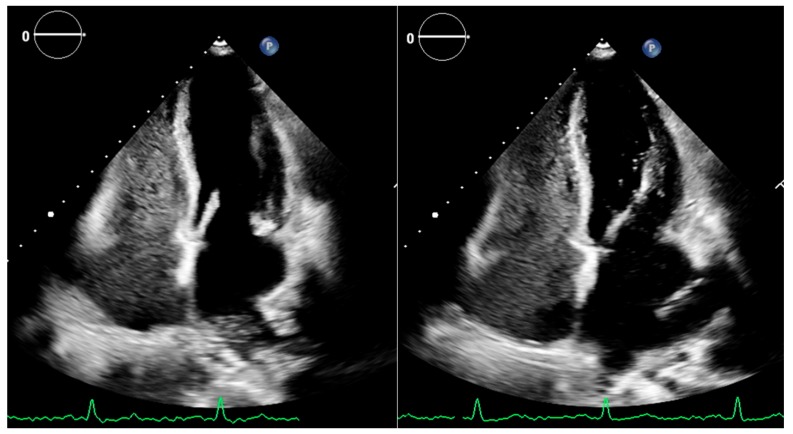
A bubble test in cirrhotic patient. Note that after 3 cardiac cycles bubbles cannot be seen within the left cardiac chambers (left figure) but are identified more than 6 cycles after contrast is seen in the right ventricle.

Contrast echocardiography with the use of saline (bubble test) is the most common method to diagnose HPS in patients with cirrhosis and/or portal hypertension and blood gas abnormalities (Figure 4). Saline-produced microbubbles can’t pass through the pulmonary circulation and thus, when seen in the left cardiac chambers they point out a right to left shunt [91]. Typically, in HPS, microbubbles are seen in the left atrium 4–10 cardiac cycles after they had been seen in the right atrium [92]. On the contrary, in endocardiac shunts, the shunt is dependent on pressure difference between right and left atrium and it is affected by breathing pattern and Valsalva maneuver. Microbubbles are typically seen within the first 3 cardiac cycles [93,94]. Because in HPS shunt worsens in the upright position, this body position may facilitate microbubbles passage in the left cardiac chambers [92]. Injection of technetium-99 m labeled macro-aggregated albumin is an alternative to bubble test, but is not widely used [95,96].

Portopulmonary hypertension (PPH) is the increase of mean pulmonary artery pressure (mPAP) in a patient with portal hypertension after other causes of pulmonary hypertension and especially elevated left ventricular filling pressure (mean pulmonary capillary wedge pressure (PCWP) > 15 mmHg) have been excluded [97]. Contrary to HPS, PPH is caused by vasoconstriction leading to increased resistance of the pulmonary circulation (>240 dynes/s/m^−5^). Traditionally, PPH is graded as mild when mPAP is 25–35 mmHg, moderate when mPAP is 35–45 mmHg and severe for mPAP values ≥ 45 mmHg [98]. However, according to recently updated clinical classification, mPaP > 20 mmHg, PCWP < 15 mmHg and pulmonary vascular resistance > 3 Wood Units are criteria for the diagnosis of pre-capillary pulmonary hypertension [99]. Because CO in cirrhotic patients is increased, and PVR is calculated as (mPap-PCWP)/CO, this definition may discriminate patients into: those with increased mPAP due to vasoconstriction and those with increased mPAP due to increased CO. Because mPAP has been shown to have a significant negative predictive role in LT candidates, exclusion of PPH is important for appropriate treatment to be initiated [100]. When increased pulmonary artery systolic pressure (PASP) cannot be justified based on findings from the assessment of the left ventricle, PPH diagnosis should be considered. Criteria for right heart catheterization are not clear because current non-invasive criteria are not accurate. Invasive measurement is justified in all patients with right ventricular systolic pressure (RVSP) ≥ 50 mmHg or findings of right heart hypertrophy [89].

## 8. Hepatopulmonary Syndrome and Portopulmonary Hypertension: Future Perspectives

Pulmonary transit time, the time-lapse of the blood to leave right ventricle and reach left atrium, has been shown to be lower in cirrhotic patients with HPS [101]. Echocardiography with contrast agents [101], angiography [102] and CMR [103] have been used for such measurement but their specificity is questionable as transit time is also affected by cardiac output [104]. CT angiography may be used to diagnose focal atrioventricular shunts [96] but it’s accuracy for HPS diagnosis remains to be assessed as small studies have shown conflicting results [105,106].

Many parameters can be determined by echocardiography (E/e’ ratio, comparison of left to right cardiac chambers, inferior vena cava analysis) for a satisfactory differentiation between pre- and post-capillary pulmonary hypertension but most of those indices have not been examined in cirrhotic patients [107]. Because E/e’ ratio is well correlated with left ventricular filling pressure, the ratio of maximal tricuspid regurgitation velocity (TRVmax) to E/e’ (echocardiographic pulmonary to left atrial-ePLAR ratio), has been shown to be significantly higher in patients with pre-capillary pulmonary hypertension [108]. As left atrial volume is increased in patients with high LV filling pressures, the ratio of right atrium to left atrium volumes has been proposed as an index of identifying patients with pre-capillary hypertension [109]. Impaired right heart ventriculo-arterial coupling as reflected by a reduced tricuspid annular plane systolic excursion (TAPSE)/PASP ratio, points towards a concomitant pre-capillary etiology of pulmonary hypertension [110]. The utility of those indices for the diagnosis of PPH remains to be assessed.

## 9. CMR Applications of Special Interest for Cardiovascular Evaluation in Cirrhotic Cardiomyopathy

### 9.1. Measurement of Volumes—Ejection Fraction

CMR measures ventricular volumes and ejection fraction noninvasively and without contrast agent [111]. Due to its high reproducibility, it is ideal for serial follow up of ventricular volumes, mass and function. Compared to echocardiography, which is an operator depended technique, with the limitations of acoustic window, CMR is operator independent and has high reproducibility [112]. The majority of CMR data in cirrhotic patients show a hyperdynamic circulation with increased cardiac chamber volume and left ventricular mass [62,113,114].

### 9.2. Myocardial Ischemia

CMR can detect ischemia by two different ways. First with observation of wall motion abnormalities (abnormal wall motion and wall thickening) using the stress factor dobutamine. Compared to stress echo, stress CMR using dobutamine has better sensitivity (86% vs. 74%) and specificity (86% vs. 70%) [115]. Second, with observation of myocardial perfusion by the first pass of a bolus of a T1-shortening contrast agent (first-pass gadolinium) injected into a peripheral vein [116]. Data acquired during intravenous vasodilator-stress (most commonly adenosine) delineate the underperfused regions associated with myocardial ischemia. The spatial resolution of CMR myocardial perfusion imaging of 2–3 mm is superior to other imaging modalities, such as nuclear techniques, so that subendocardial ischemia can be more reliably identified [116]. Its high diagnostic accuracy in coronary heart disease and superiority over SPECT was established by the Clinical Evaluation of MAgnetic Resonance imaging in Coronary heart disease (CE-MARC) study [117]. Myocardial perfusion abnormalities can be attributed to epicardial CAD or microcirculation disorders and is frequently abnormal in cirrhotic patients [118]. A negative for ischemia CMR stress examination in LT candidates has been shown to have an almost 100% CAD event free survival at 12 months [119].

### 9.3. Detection of Blunted Inotropic Response to Pharmacologic Stress

CCM is characterized by an impaired cardiac pharmacological response that can be detected with magnetic resonance myocardial stress testing. Krag et al. showed that inotropic response to stress is normal in patients with early cirrhosis [120]. CMR deformation analysis parameters may be more sensitive in identifying abnormalities in inotropic response to stress than conventional methods and can demonstrate impaired longitudinal strain in cirrhotic patients [121].

### 9.4. Fibrosis Detection/Late Gadolinium Enhanced (LGE) Imaging

Following acute ischemic injury, the myocardial distribution volume of gadolinium is increased, due to sarcrolemmal rupture and abnormal wash-out kinetics. This method is referred in the literature as late gadolinium enhanced CMR (LGE) and is the gold standard for the in vivo assessment of myocardial scar [122]. CMR can detect infarction in as little as 1 cm^3^ of tissue, substantially less than other in vivo methods, such as conventional echocardiography and nuclear techniques even when only the subendocardium is involved [123,124]. The CMR extent of scar predicts the potential for functional recovery after revascularisation [125] and patient prognosis [126]. However, even a small area of LGE (<2% of LV mass) was associated with a greater than 7-fold increase in risk for a major adverse cardiac event [127].

In case of CCM, late gadolinium enhancement has a diffuse myocardial distribution in CMR images with the pattern of myocarditis [113]. High LGE related with alcoholic etiology and high cardiac index. Patients with very high levels of NT-pro-BNP were the ones with high LGE. These data indicate that diffuse fibrosis in cirrhotic patients is seen when cirrhotic cardiomyopathy is more severe, a feature which can be quantified by CMR.

### 9.5. Iron Deposition Assessment

“T2-star” technique, assessed by CMR, is a non-invasive method for measuring liver and cardiac iron deposition [128]. Values lower than 20 ms are indicative of cardiac iron overload. Iron deposition in patients with liver disease is common in patients with hereditary hemochromatosis or transfusion dependent beta-thalassemia [128,129]. Cirrhosis can also lead to cardiac iron overload found in autopsy examinations [130,131]. Patients with cardiac iron overload have more advanced liver disease [132]. Lewin et al. used T2* technique to quantify cardiac iron deposition in LT candidates. T2* values less than 20 ms related to MELD score of more than 25 and systolic dysfunction. Furthermore, T2* values less than 15 ms were predictive of heart failure after LT and showed a significant hazard ratio of 3.85 when compared to patients with T2* ≥ 20 ms [133].

### 9.6. Tissue Characterization and Parametric Imaging

CMR has the capability to characterize myocardial tissue using T_1_ and T_2_ mapping techniques. Quantitative T_1_ imaging, in particular, can be used to calculate the myocardial extracellular volume fraction (ECV), a measure of microscopic myocardial remodeling that has been associated with underlying diffuse fibrosis [134]. ECV was associated with disease severity (Child Pugh class) and worse transplant-free survival [135]. ECV in cirrhotic patients decreased at 1 year after transplantation, suggesting normalization of the LV systolic function and a decrease in diffuse myocardial fibrosis [114]. These findings may facilitate to establish diagnostic criteria for early diagnosis of CCM in LT candidates.

### 9.7. Limitations of CMR and Application in LT Candidates

Limitations of CMR include lack of availability, high cost, high expertise level needed for accurate diagnosis, awareness of referring physicians about the applications of the technique, claustrophobia, metallic clips, pacemakers (unless CMR compatible) and defibrillator [136]. Even though, according to Reddy et al. CMR was feasible and 45 out of 51 patients completed the examination which lasted 72 min and included the exclusion of hepatocellular carcinoma [137].

## 10. Conclusions

Cardiovascular dysfunction in cirrhotic patients without a preexisting cardiac disease is usually referred to as CCM, a condition characterized by high cardiac output and usually preserved systolic function at rest. Careful evaluation of diastolic function and identification of blunted response to adrenergic stimulation may unmask this clinical condition. Newer imaging modalities such as STE, as well as CMR may contribute to improved diagnostic approach of cirrhotic patients, but they are not used in the clinical practice nor are they included in the diagnostic criteria. More data are needed to investigate their clinical utility and limitation for the LT candidates. Given the increased prevalence of metabolic syndrome, NASH associated cirrhosis has become more frequent increasing the proportion of cirrhotic patients with CAD. Standard protocols have low sensitivity for CAD, giving prominence to the need for evaluation of the prognostic significance of mixed stress echo protocols and stress CMR. Other cardiovascular abnormalities in LT candidates include HPS and PPH, which are usually diagnosed with conventional echocardiography. We believe that a multidisciplinary team including both hepatologists and cardiologists can take advantage of the multimodality imaging approach to improve clinical outcomes of LT candidates. Such approach should be reflected in future diagnostic criteria and guidelines.

## Figures and Tables

**Table 1:** **Cirrhotic** cardiomyopathy diagnostic criteria according to the World Gastroenterology Organisation (Montreal 2005).

Cirrhotic patient with	Abnormal contractile response to stress Diastolic dysfunction Absence of another clinically significant cardiopulmonary Disease
Systolic function (at least 1)	⮚ Blunted increase in cardiac output with exercise, volume challenge or pharmacologic stimuli. ⮚ Resting left ventricular ejection fraction (LVEF) < 55%
Diastolic function (at least 1)	⮚ E/A ratio < 1 (age corrected) ⮚ Prolonged mitral deceleration time (DT > 200 ms) ⮚ Prolonged isovolumetric relaxation time (>80 ms)
Supportive criteria	⮚ Abnormal chronotropic response to stress ⮚ Electromechanical uncoupling ⮚ Dysychrony ⮚ Prolonged QTc interval ⮚ Enlarged left atrium ⮚ Increased left ventricular mass ⮚ Increased BNP or proBNP ⮚ Increased troponin I.
